# Anatomic single- and double-bundle ACL reconstruction both restore dynamic knee function: a randomized clinical trial—part II: knee kinematics

**DOI:** 10.1007/s00167-021-06479-x

**Published:** 2021-02-22

**Authors:** Scott Tashman, Payam Zandiyeh, James J. Irrgang, Volker Musahl, Robin Vereeke West, Neha Shah, Freddie H. Fu

**Affiliations:** 1 Steadman Philippon Research Institute, Vail, CO USA; 2 Department of Physical Therapy, University of Pittsburgh, Pittsburgh, PA USA; 3 UPMC Center for Sports Medicine, Pittsburgh, PA USA; 4 Inova Sports Medicine, Fairfax, VA USA; 5 Department of Orthopaedic Surgery, University of Pittsburgh, 3471 Fifth Avenue, Pittsburgh, PA 15213 USA

**Keywords:** Anterior cruciate ligament, ACL reconstruction, Anatomic double-bundle, Anatomic single-bundle, Kinematics, Randomized clinical trial

## Abstract

**Purpose:**

Compare side-to-side differences for knee kinematics between anatomic single-bundle (SB) and anatomic double-bundle (DB) ACLR during downhill running at 6 and 24 months post ACLR using high-accuracy dynamic stereo X-ray imaging. It was hypothesized that anatomic DB ACLR would better restore tibio-femoral kinematics compared to SB ACLR, based on comparison to the contralateral, uninjured knee.

**Methods:**

Active individuals between 14 and 50 years of age that presented within 12 months of injury were eligible to participate. Individuals with prior injury or surgery of either knee, greater than a grade 1 concomitant knee ligament injury, or ACL insertion sites less than 14 mm or greater than 18 mm were excluded. Subjects were randomized to undergo SB or DB ACLR with a 10 mm-wide quadriceps tendon autograft harvested with a patellar bone block and were followed for 24 months. Dynamic knee function was assessed during treadmill downhill running using a dynamic stereo X-ray tracking system at 6 and 24 months after surgery. Three-dimensional tibio-femoral kinematics were calculated and compared between limbs (ACLR and uninjured contralateral) at each time point.

**Results:**

Fifty-seven subjects were randomized (29 DB) and 2-year follow-up was attained from 51 (89.5%). No significant differences were found between SB and DB anatomic ACLR for any of the primary kinematic variables.

**Conclusions:**

Contrary to the study hypothesis, double-bundle reconstruction did not show superior kinematic outcomes compared to the single-bundle ACLR. While neither procedure fully restored normal knee kinematics, both anatomic reconstructions were similarly effective for restoring near-normal dynamic knee function. The findings of this study indicate both SB and DB techniques can be used for patients with average size ACL insertion sites.

**Level of evidence:**

Level I

## Introduction

Anterior cruciate ligament (ACL) rupture is a common knee injury, especially in high-demand sports. The most common surgical treatment is ACL reconstruction (ACLR) using tendon autografts, with the primary objective to improve knee stability and overall function, minimize further damage to the knee and reinstate anatomical, mechanical constraints imposed by the native ACL. Anatomically, the ACL can be divided into antero-medial (AM) and postero-lateral (PL) bundles which, when reconstructed, have shown improve anterior–posterior and rotation knee laxity in reconstructed knees [17, 30]. In vivo studies have shown that conventional single-bundle (SB) ACLR fails to restore rotational knee function during dynamic, functional loading [20, 23]. A growing number of studies have also reported high rates of knee osteoarthritis occurring 5–20 years after ACL injury/surgery [16, 23] with abnormal knee kinematics suggested as a contributing factor [6]. This had incentivized the advancement of anatomic “double-bundle” (DB) surgical techniques for reconstructing both the AM and PL bundles of the ACL to better restore normal knee kinematics.

There have been a limited and inconclusive number of studies comparing the results of SB versus DB reconstruction. Some clinical studies regarding the restoration of normal knee kinematics have reported minimal differences in translational and rotational laxity [5, 18, 31] while others have identified reduced rotational knee laxity for DB compared to SB techniques [14]. These previous studies have generally assessed the efficacy of ACLR for restoring rotational knee laxity based on passive laxity tests, with “so” knees exhibiting minimal differences in laxity compared to the contralateral, uninjured knee. It is essential to measure knee kinematics during dynamic, functional activities, particularly to identify abnormal joint mechanics that may occur during everyday life and cumulatively could lead to long term joint degeneration. Furthermore, there is limited evidence suggesting that DB reconstruction leads to superior joint stability under in vivo, dynamic loading conditions [24]. Superior rotational stability for DB versus SB ACLR has been reported during cutting movements [9]. Others have reported no significant differences between SB and DB ACLR for rotational stability during stair ascent/descent [27] or gait. Given the known limitations of the video-motion analysis techniques employed, it is unclear from these studies whether SB and DB ACLR truly produce the same kinematic outcomes or if the measurement techniques have insufficient sensitivity to detect small (but potentially clinically significant) differences.

There are likely many factors that have contributed to the inconsistent findings of studies comparing SB versus DB reconstruction. There have been few randomized trials, and there has been considerable variability in surgical technique, tunnel locations, and graft types across studies. Parallel with the development of DB techniques; there has been a greater focus on placing grafts (both SB and DB) more “anatomically” (i.e., within the native ACL footprints), to more closely replicate native ACL anatomy [7]. Anatomic SB reconstruction (performed using visualization of the femoral insertion site via the medial portal) provides reduced anteroposterior and rotational laxity compared to SB techniques that utilize trans-tibial drilling of the femoral tunnel [10]. The variability of tunnel locations, fixation and tensioning methods and graft type across studies, often combined with under-reporting of specific operative methods [28] has further clouded understanding of the effects of incorporating one versus two bundles on knee function after ACLR.

To overcome many of the limitations of previous studies, a prospective, double-blind, randomized clinical trial was designed to compare dynamic knee function after SB versus DB ACLR [12]. The goal of this study was to compare knee kinematics during dynamic, functional activities between SB-reconstructed, DB-reconstructed, and contralateral (uninjured) limbs at 6 and 24 months after ACL reconstruction. It was hypothesized that anatomic DB ACLR would better restore tibio-femoral kinematics compared to SB ACLR, based on comparison to the contralateral, uninjured knee. Secondarily it was hypothesized that limb-to-limb differences would decrease more over time in the DB group compared to the SB group.

## Materials and methods

The kinematic results reported here represent additional outcome measures acquired as part of a comprehensive clinical trial comparing SB and DB ACL reconstruction. Detailed study design, recruitment, randomization and surgical details, as well as results for clinical outcomes measures, are provided in Part 1 [refer to part 1 manuscript here]. A brief summary is provided below.

### Participants

This study was approved by University of Pittsburgh Institutional Review Board for Biomedical Research (PRO09020493) and registered on ClinicalTrials.gov (NCT01319409). Participants were recruited from the clinical practices of the authors between March 2011 and December 2012. Individuals between 14 and 50 years of age with a complete tear involving both bundles of the ACL with or without medial or lateral meniscus injury were eligible if they presented for surgery within 12 months of injury, participated in at least 100 h of level 1 (e.g., football, basketball or soccer) or 2 (e.g., racquet sports, skiing, manual labor occupations) activities in the year prior to injury and had tibial and femoral arthroscopically verified ACL insertion site widths between 14 and 18 mm. Exclusion criteria included prior injury or surgery of the ipsilateral or contralateral knee, greater than a grade 1 concomitant knee ligament injury, a full-thickness cartilage injury, open femoral or tibial growth plates, inflammatory or other forms of arthritis, any other injury or condition involving the lower extremity affecting the individual’s ability to participate in Level 1 or 2 activities or plans to move from the region within the study follow-up period. Females who were pregnant or had plans to become pregnant within two years were excluded. Since the participant’s quadriceps tendon was used to reconstruct the ACL, individuals were also excluded if the preoperative MRI showed intra-tendinous degeneration of the quadriceps tendon or thickness of the quadriceps tendon of less than 7 mm.

### Surgical procedures

**Fig. 1:**
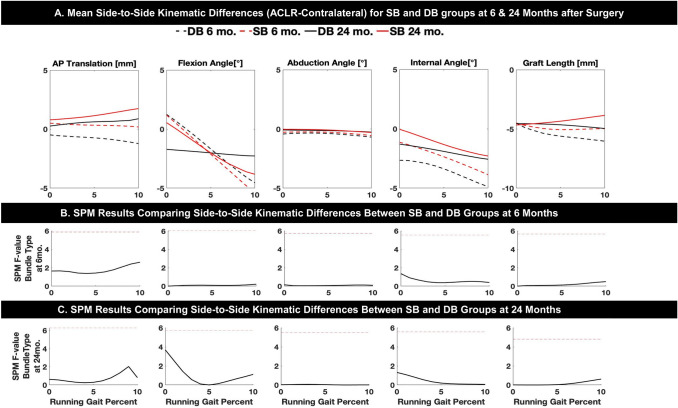
**a** Mean side-to-side differences (SSD; ACLR-contralateral) in tibio-femoral kinematics (anterior tibial translation, flexion, abduction, internal rotation and functional graft length) during the stance phase of downhill running (first 10% of the running cycle) for SB and DB groups at 6 and 24 months after surgery. **b**, **c** Statistical pattern matching (SPM) results comparing SSD between SB and DB at 6 (**b**) and 24 (**c**) months after surgery. Dashed red line represents the *F *value threshold corresponding to statistical significance (*p* < 0.05). No significant differences were found for side-to-side differences between SB and DB

All surgeries were performed using standardized procedures for anatomic ACLR. The procedures for anatomic DB ACLR and SB ACLR have been published [22]. To avoid graft type as a confounding factor, a 10 mm-wide autograft quadriceps tendon with a patellar bone block was used in all cases [12]. For DB ACL reconstruction, the 10 mm-wide quadriceps tendon graft was split, leaving the bone block as one, into grafts to reconstruct the anteromedial (AM) bundle and the posterolateral (PL) bundle. To minimize bias with graft harvest, the harvest was completed before the subject was randomized into the SB or DB group. For DB ACLR, one femoral tunnel in the center of the femoral insertion site and two tibial tunnels corresponding to the insertions of the AM and PL bundles were created to reproduce the normal insertion site anatomy. For SB ACLR, one femoral and one tibial tunnel were created in the center of the femoral and tibial insertion sites, respectively (see Fig. 1a, b). If necessary, meniscus repair or meniscectomy or chondroplasty was performed.

### Post-operative rehabilitation

All participants underwent a standardized post-operative rehabilitation program, supervised by a physical therapist, as previously described [11]. The essential features included an early emphasis on control of pain and swelling, restoring full passive knee extension symmetrical to the non-involved knee, maintaining patellar mobility, regaining quadriceps strength and protected weight-bearing. Once participants achieved full weight bearing, they progressed to progressive resistance exercises as tolerated and balance and perturbation activities were initiated. Approximately 4 months post-surgery, participants progressed to running, agility and plyometric drills and return to sports activities as tolerated, with unrestricted return to sports expected 9–12 months post-surgery. Return to sport protocol was based on sport readiness tests developed at our institution [13].

### Data collection

Two functional tasks were selected for evaluating knee kinematics. Walking is the dominant joint loading activity for most individuals and was assessed at 1.3 m/s (normal adult gait speed). Downhill running (3 m/s) was selected as it challenges knee stability without placing individuals at significant risk for injury and has been previously shown to reveal differences between ACL-reconstructed and uninjured knees [26]. Use of a treadmill assured consistent walking and running speeds within and across participants and facilitated maintaining the knee joint within the field of view of the imaging system. Data were collected 6 and 24 months post-surgery. This protocol has previously shown to have high retest reliability per previous studies [25]. Actual radiographic exposure was reduced to the minimum level necessary to obtain adequate image quality.

Participants wore athletic footwear during testing and were provided sufficient time to accommodate to treadmill locomotion prior to data collection. Three repetitions were performed for each task (walking, downhill running) on each leg (ACL-reconstructed, contralateral), for a total of 12 trials, with limb order randomized. Sufficient time between trials was provided to minimize fatigue. Exposures were timed to include data from just before foot-strike through mid-stance, to capture kinematics during the period of highest loading of the knee.

Three-dimensional position and orientation of the tibia and femur were determined using a validated model-based tracking system, as previously described [1]. Briefly, DSX images were corrected for distortion and intensity non-uniformity. The 3D geometry of the DSX system was determined using a 12-marker phantom and a direct linear transformation (DLT) calibration algorithm. Volumetric and surface models of the distal femur and proximal tibia were produced via manual segmentation of the CT scans using commercially available software (Mimics, Materialize, Inc.). Anatomic landmarks were identified from the bone surface models to define standard clinical axes, as previously described [2]. Three-dimensional locations of bone tunnel centers were determined at the tunnel apertures. Volumetric models (for model-based tracking) were built from the same segmented images, with all grayscale/internal features preserved. Models were interpolated using an edge/feature-based algorithm to generate 0.25 mm^3^ voxels. Three-dimensional motions of the tibia and femur were calculated from the DSX image sequences, using a model-based tracking technique that optimized the correlation between the DSX radiographs and digitally reconstructed radiographs created from the volume-rendered CT model [32].

Three-axis rotations of the tibia relative to the femur were calculated for each trial using the ordered-rotation conventions proposed by Grood and Suntay [8]. Three-dimensional translations were determined relative to ACL graft origin/insertion locations on the tibia and femur (defined as the centers of the graft tunnel apertures from CT-based subject-specific 3D bone models) and expressed in an anatomical coordinate system fixed to the tibia. Functional ACL graft length was defined by the 3D distance from ligament/graft origin to insertion. For subjects with DB reconstructions, location of the native ACL tibial insertion was estimated as the average between the AM and PL tunnel centers. For the contralateral, ACL-intact knee, the ACL origin and insertion footprints were determined from MRI as previously described and validated [4]. The centroids of the footprints were then used for AP translation and ACL length calculations. Kinematics data timing was normalized to the percentage of the movement cycle, with 0% corresponding to initial foot-strike and 100% the second foot-strike of the same foot.

### Data and safety monitoring

An independent Data and Safety Monitoring Board (DSMB) was appointed by the funding agency (NIH) to oversee the conduct of the study, participant safety, data integrity of the data and validity of the study results. Adverse events were monitored continuously throughout the study and were reviewed by the investigators and DSMB to determine severity and relationship with the research intervention and procedures. No formal interim analyses were conducted during the trial.

### Statistical analysis

A Statistical Parametric Model analysis (SPM) [21] was performed to compare the kinematic differences for within-subjects factors of time (6 vs. 24 months) and limb (ACLR vs. Contralateral), and the between-subjects factor of surgical technique (SB vs. DB). Statistical parametric model analysis is gaining popularity in biomechanics research because it is specifically designed to compare and detect pointwise differences between trajectories (e.g., kinematic motion curves). Each SPM test results in a test statistic curve (in this case, the t statistic as a function of time), with overall differences between the curves assessed statistically using random field theory (RFT). Significance at each individual timepoint of the curves is only assessed if the RFT test for the entire curve is significant. This method is superior to more conventional statistical approaches for comparing joint trajectories (e.g., analysis of variance of selected points, means, minimums, maximums) as it mitigates bias in hypothesis testing that results from analysis of selected data values [21] and also addresses issues of multiple comparisons (a classic problem in biomechanics research) in a theoretically robust manor.

The differences between SB and DB reconstruction for the following tibio-femoral kinematic measures were calculated and used for analysis: anterior translation (AP), medial translation (ML), flexion angle (Flex), abduction angle (AbAd), internal rotation angle (IE) and anatomic ACL length (ACL).

## Results

From original screening of 249 participants between March 2011 and December 2012, 157 individuals were not eligible to participate in the study. Of the 92 eligible participants, 32 declined to consent and three provided consent but were not randomized before the study was terminated. The remaining 57 participants were randomized to SB (*n* = 28) or DB (*n* = 29) ACLR. Demographics for the SB and DB groups were similar (Table 1). Six patients (2 DB, 4 SB ACLR) were lost to follow-up resulting in an overall follow-up rate of 89.5%. For more details on recruitment and followup (including a CONSORT Flow Chart), please refer to the companion paper describing this study.

**Table 1:** Demographic summary

	Double bundle (*n* = 29)	Single bundle (*n* = 28)
Age (years, mean ± SD)	23.1 ± 9.2	20.3 ± 4.3
Male (*n*, %)	18, 62.1%	20, 71.4%
Weight (lbs, mean ± SD)	170.8 ± 28.2	167.5, 28.1
Height (inches, mean ± SD)	69.1 ± 3.4	68.9 ± 3.6
Body mass index (kg/m^2^, mean ± SD)	25.1 ± 3.4	24.7 ± 2.7

Data were collected from all subjects for both walking and downhill running. While overall kinematic trends were similar, statistical results for SB versus DB comparisons were the same for both activities and differences between the ACLR and contralateral limbs were consistently larger for downhill running. Thus, in the interests of brevity, only the downhill running results are presented.

Figure 1 shows the average kinematic outcomes for SB and DB surgical techniques during downhill running at 6 and 24 months after surgery. All knee kinematic measures were similar between the two surgical groups (SB and DB). There were no statistically significant differences between SB and DB groups in side-to-side (ACLR-contralateral) measures of knee kinematics in any movement plane for either walking or downhill running. Also, no significant differences were identified between SB and DB for kinematic changes within the ACLR limbs over time (from 6 to 24 months after surgery), as confirmed via SPM results for interactions between time after surgery and surgical technique.

**Fig. 2:**
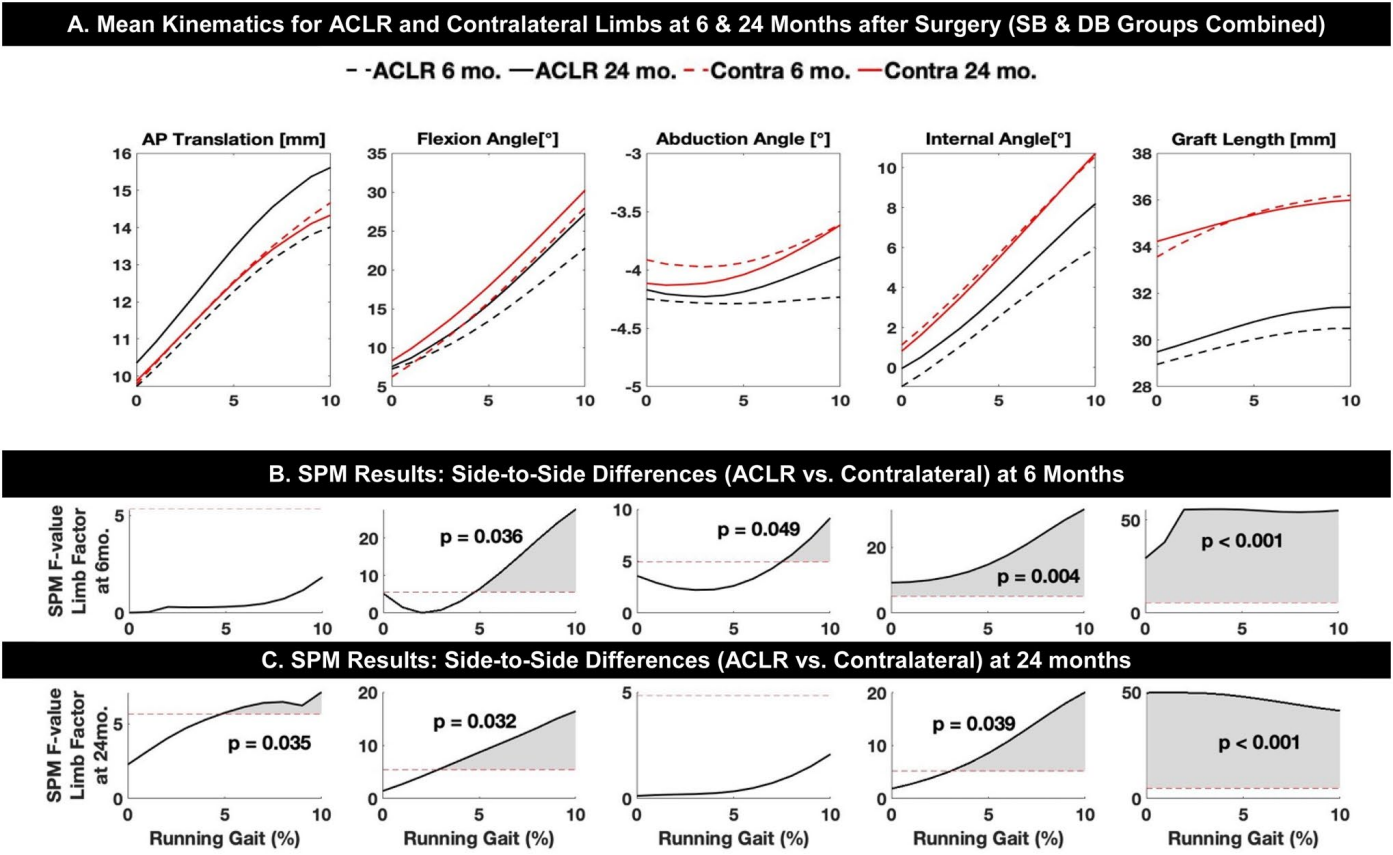
**a** Mean tibio-femoral kinematics (anterior tibial translation, flexion, abduction, internal rotation and functional graft length) during the stance phase of downhill running (first 10% of the running cycle) of the combined groups for ACLR and Contralateral (uninjured) limbs at 6 and 24 months after surgery. **b**, **c** Statistical parametric mapping (SPM) results for comparisons over time and between limbs. *p *values listed represent significant differences between entire curves. Dashed red line represents the *F* value threshold corresponding to statistical significance (*p* < 0.05); gray-shaded regions are where *F *values exceed this threshold, illustrating periods of time where curves are significantly different. **b** SPM results for differences between ACLR and Contralateral limbs 6 months after surgery. **c** SPM results for differences between ACLR and Contralateral limbs 24 months after surgery

Given the absence of any significant differences between the SB and DB groups, data from both groups were combined for subsequent analyses (*n* = 51 subjects total). For the combined SB and DB groups, significant changes were identified in knee kinematics from 6 versus 24 months after surgery (Fig. 2). The flexion angle in the ACLR limb increased over time for 4–10% of the running cycle (*p* = 0.018; 1.6°–4.4°). Internal rotation was significantly increased at 24 months for 8–10% of the running cycle (*p* = 0.049; 1.7°–2.2°), reducing the amount of abnormal external rotation relative to the contralateral limb. Anterior translation (*p* = 0.022; 1.2–1.6 mm) and graft elongation (*p* = 0.046; 0.8–0.9 mm) increased over time, and these changes were significant from 5 to 10% of the running gait. Abduction did not change significantly over time.

Across both groups, knee kinematics differed between ACLR and contralateral limbs (BLINDED, B, C) 6 months after surgery, the ACLR limb was more adducted compared to the contralateral limb (*p* = 0.049; 0.4°–0.6°) but the difference was no longer significant at 24 months. The ACLR limb was more externally rotated throughout the early stance phase at 6 months (*p* = 0.004; 2.1°–4.6°). At 24 months, this difference was smaller but still significant from 3 to 10% of the running cycle (*p* = 0.039; 1.6°–2.5°). While not significantly different from the contralateral limb at footstrike, the ACLR limb was less flexed than the contralateral limb at 5–10% of the running cycle at 6 months (*p* = 0.036; 2.4°–5.2°) and 3–10% of the running cycle at 24 months (*p* = 0.032; 2.1°–3.0°). Last, the graft length was shorter compared to the native ACL length at six (*p* < 0.001; 4.6–5.7 mm) and 24 months (*p* < 0.001; 4.5–4.7 mm) throughout early stance.

## Discussion

The primary and most important finding of this study was that in knees with ACL insertion sites that ranged from 14 to 18 mm, there were no detectable differences in tibio-femoral kinematics for DB ACLR compared to SB reconstruction. Additionally, no evidence was found that DB ACLR had notable impact in comparison to SB ACLR on changes in knee kinematics from 6 to 24 months after ACLR. Thus, DB reconstruction as performed for this study in patients appears to have minimal benefit over anatomic SB reconstruction as was originally hypothesized.

Previous studies using video-motion analysis and skin surface markers were unable to detect kinematic differences between SB and DB [19, 27] because of the significant errors due to skin motion artefact [25]. The measurement accuracy and precision of the DSX system employed for this study has validated translational and rotational precisions greater than traditional video-motion analysis [1]. Therefore, the measurements in this study were suitably precise and accurate to detect even subtle differences between the DB and SB methods to reconstruct the ACL.

The ultimate goal for mechanical restoration of ACL function is to replicate the native ACL insertion site size and geometry as closely as possible, since deviation of the graft tunnels from the native ACL insertion can negatively affect knee mechanics during functional movements [29]. ACL insertion site anatomy is highly variable [15] creating challenges with central positioning of a single, large circular tunnel within the ligament footprint for some individuals. Surgeon judgement is recommended to determine the most appropriate surgical technique on a case-by-case basis, considering individual anatomy.

While anatomic SB and DB reconstruction resulted in similar knee kinematics, neither completely restored normal dynamic knee function in comparison to the contralateral normal knee. This study detected the presence of residual external tibial rotation in the ACLR limb during running (at 6 and 24 months), similar to previous findings by Tashman et al. [25] and others, and may result in abnormal loading of articular cartilage, potentially leading to osteoarthritis [3]. It is not yet clear what magnitude of internal rotation differences are clinically meaningful. Longer follow-ups are required to determine whether there is a relationship between the rotational abnormalities identified here and the associated risk for developing osteoarthritis.

An important finding was that the length of the reconstructed grafts was shorter on average by 4–5 mm compared to the native ACL. Additionally, the mechanical stiffness of the harvested graft (i.e., the quadriceps tendon) is greater than the native ACL. A graft that is both shorter and stiffer than the native ACL likely results in over-constraint of the joint, leading to a more externally rotated tibia. The observed reduction in abnormal external tibial rotation over time in the current study (side-to-side difference was reduced by 1.2° from 6 to 24 months) was coincident with an increase in functional graft length and anterior tibial translation. These changes might be related to biological, vascular, and cellular remodeling, leading to a gradual reduction of stiffness and/or stretching of the ACL graft over time.

It is notable, however, that no significant differences were identified between ACLR and contralateral limbs for knee ab/adduction. This is in contrast to the previous study (using similar methods) [25] where significantly increased knee adduction was identified in the ACLR limb during downhill running. The primary difference from the present study is that a “conventional” (non-anatomic) surgical technique was used for ACL reconstruction. While no definitive conclusions can be drawn from this observation, the comparative results of these two studies suggest that anatomic reconstruction (whether SB or DB) may lead to improved kinematics compared to non-anatomic techniques.

Studying the side-to-side flexion angle differences may provide valuable insights into the adaptation mechanism of the ACLR limb. Over time, the range of knee flexion angle differences between limbs was reduced from 2.4° to 5.2° at 6 months to 2.1°–3° at 24 months. Reduction in side-to-side differences and consequently deeper flexion of the ACLR limb during the stance phase is desirable since it represents an improvement in side-to-side symmetry and may contribute to improved balance in functional loading between the limbs. Increased knee flexion under eccentric contraction of the quadriceps may also help to absorb impact forces in early stance phase, protecting the knee from excessive loading.

Clinically, either SB or DB ACLR can be recommended for individuals with tibial insertion sites between 14 and 18 mm in size as both grafts appear to be equally effective for restoring near-normal knee kinematics. However, there are several benefits to SB ACLR, namely shorter operation time, lower cost, less technical complexity, and easier revision. Moreover, DB reconstruction requires more fixation points, more tunnels and increased risk of osteonecrosis and chondrolysis. Therefore, SB reconstruction may be the preferred approach for patients with average size ACL insertion sites.

The study has limitations. No information is available on the kinematic behavior before the ACL surgery or a healthy control group.  The inclusion of older patients (age 14–50) created an inhomogeneous study group; however, the inclusion criteria was strict in excluding older patients with concurrent knee diagnoses including grade 3 cartilage lesions or greater.

## Conclusion

Clinically, either SB or DB ACLR can be recommended for individuals with tibial insertion sites between 14 and 18 mm in size as both grafts appear to be equally effective for restoring near-normal knee kinematics.
